# Previous miscarriage and the subsequent risk of preterm birth in Scotland, 1980–2008: a historical cohort study

**DOI:** 10.1111/1471-0528.13276

**Published:** 2015-01-28

**Authors:** C Oliver-Williams, M Fleming, AM Wood, GCS Smith

**Affiliations:** a Department of Public Health and Primary Care, University of CambridgeCambridge, UK; b Information Services Division, NHS National Services ScotlandEdinburgh, UK; c Department of Obstetrics and Gynaecology, NIHR Biomedical Research Centre, University of CambridgeCambridge, UK

**Keywords:** Miscarriage, premature birth, spontaneous termination of pregnancy

## Abstract

**Objective:**

To determine whether the relationship between previous miscarriage and risk of preterm birth changed over the period 1980–2008, and to determine whether the pattern varied according to the cause of the preterm birth.

**Design:**

Linked birth databases.

**Setting:**

All Scottish NHS hospitals.

**Population:**

A total of 732 719 nulliparous women with a first live birth between 1980 and 2008.

**Methods:**

Risk was estimated using logistic regression.

**Main outcome measures:**

Preterm birth, subdivided by cause (spontaneous, induced with a diagnosis of pre-eclampsia, or induced without a diagnosis of pre-eclampsia) and severity [extreme (24–28 weeks of gestation), moderate (29–32 weeks of gestation), and mild (33–36 weeks of gestation)].

**Results:**

Consistent with previous studies, previous miscarriage was associated with an increased risk of all-cause preterm birth (adjusted odds ratio, aOR 1.26; 95% confidence interval, 95% CI 1.22–1.29). This arose from associations with all subtypes. The strongest association was found with extreme preterm birth (aOR 1.73; 95% CI 1.57–1.90). Risk increased with the number of miscarriages. Women with three or more miscarriages had the greatest risk of all-cause preterm birth (aOR 2.14; 95% CI 1.93–2.38), and the strongest association was with extreme preterm birth (aOR 3.87; 95% CI 2.85–5.26). The strength of the association between miscarriage and preterm birth decreased from 1980 to 2008. This was because of weakening associations with spontaneous preterm birth and induced preterm birth without a diagnosis of pre-eclampsia.

**Conclusions:**

The association between a prior history of miscarriage and the risk of preterm birth declined in Scotland over the period 1980–2008. We speculate that changes in the methods of managing incomplete termination of pregnancy might explain the trend, through reduced cervical damage.

## Introduction

Miscarriage, the spontaneous loss of a pregnancy before 24 weeks of gestation, affects up to 15% of known pregnancies, with the majority of miscarriages occurring in the first trimester (before 13 weeks of gestation).^1^ It is well established that a history of miscarriage is associated with an increased risk of preterm birth in the next continuing pregnancy.^2^ With increasing rates of preterm birth in almost all countries with reliable data,^3^ and the greater risk of neonatal death and disability that arises from prematurity, it is important to identify potential causes of prematurity.

One potential mechanism that could explain an association between previous miscarriage and risk of preterm birth is a weakening of the cervix as a result of damage from the surgical management of miscarriage. This mechanism has been postulated to explain the association between previous therapeutic pregnancy termination and increased risk of preterm birth.^4^ Interestingly, changes in the methods used to achieve therapeutic pregnancy termination in Scotland have occurred over the last 30–40 years, and have been paralleled by a declining strength of association between previous pregnancy termination and subsequent risk of preterm birth.^5^ Similarly, the management of miscarriage has changed over recent years, with an increased use of medical methods.^6^

A recent meta-analysis found an increased risk of preterm birth in women with one previous miscarriage (odds ratio, OR 1.43; 95% confidence interval, 95% CI 1.05–1.56), and that the risk was greater still in women with multiple previous miscarriages (OR 2.27; 95% CI 1.98–2.81);^2^ however, there was evidence of between-study heterogeneity (significant differences between the studies.) This heterogeneity may have arisen from differing methods of management between the studies, or through including studies from different time periods.

The hypothesis that cervical damage arising from surgical management is causally associated with the subsequent risk of preterm birth makes two predictions: (1) that there should be a dose–response relationship between the number of previous miscarriages and the risk of preterm birth; and (2) that there should be a decline in the strength of the association over recent years. The present study used logistic regression to test these hypotheses using data collected from across Scotland.

## Methods

### Study populations

Two databases were used in the analysis. The Scottish Morbidity Record 02 (SMR02) was linked with the Scottish Stillbirth and Infant Death Survey (SSBIDS). SMR02 records the clinical and demographic characteristics and outcomes of all patients discharged from Scottish maternity hospitals, and SSBIDS classifies all perinatal deaths in Scotland. Approval for the record linkage was provided by the National Health Service (NHS) National Services Scotland Privacy Advisory Committee (PAC). We studied liveborn, first births that occurred at or after 24 weeks of gestation between 1980 and 2008.

### Definitions

Miscarriage was defined as a pregnancy that ended spontaneously with the loss of a non-registerable fetus (defined as <28 weeks of gestation until 30 September 1992, and <24 weeks of gestation thereafter), and was self-reported at the first antenatal visit.

Preterm birth was defined as birth before 37 weeks of gestation. It was subdivided into spontaneous and induced preterm birth. Induced preterm births were defined as those where there was either a pre-labour caesarean section or there was a documented method of induction of labour. Induced preterm birth was then separated by aetiology, into induced preterm delivery with a diagnosis of pre-eclampsia and without a diagnosis of pre-eclampsia. Pre-eclampsia was identified through the International Classification of Diseases (ICD) codes 642.4 or 642.5 (ninth revision), or codes O140, O141, or O149 (tenth revision).

Socio-economic deprivation was measured using the Carstairs socio-economic deprivation score, a scoring system based on census data of car ownership, unemployment, overcrowding, and social class, within postcode sectors of residence that contain approximately 16 000 people.^7^ There were seven categories (1, least deprived; 7, most deprived). Height, measured in centimetres, was evaluated at the first antenatal visit. Smoking during pregnancy, history of pregnancy termination, and marital status were self-reported at the first antenatal visit. Smoking was defined as current, never, or ex-smoker. Marital status was defined as married or other, which included co-habiting, divorced, widowed, and single. Maternal age was defined as the mother's age on the day of her child's birth.

### Statistical analyses

Continuous variables were summarised by median and interquartile range; comparisons across miscarriage groups were conducted by Kruskal–Wallis test. Categorical variables were summarised by number and percentage, and groups were compared using the chi-square test.

Logistic regression was used to model the risk of preterm birth with and without adjustment for maternal characteristics. Cox regression was used to determine whether associations with preterm birth subtypes varied across the gestational age range of 24–36 weeks of gestation.^8^ The ‘time to event’ was defined as gestational age at birth, preterm birth subtypes that were not the outcome of interest were not included, and deliveries beyond 36 weeks of gestation were treated as censored as events. The proportional hazards assumption was assessed using the Grambsch–Therneau test.^9^ Where there was strong evidence of non-proportionality, the births were stratified by gestational age at birth in order to assess the strength of the association in extremely preterm births (24–28 weeks of gestation), very preterm births (29–32 weeks of gestation), and moderately preterm births (33–36 weeks of gestation).

To demonstrate the consistency of associations, a history of previous miscarriages was treated as a binary exposure (any previous miscarriages versus no previous miscarriages), a categorical exposure (0, 1, 2, and 3+ miscarriages), and a continuous variable (truncated at 3 because of the small numbers of women with more than three previous miscarriages).

Multivariate analyses adjusted for year of delivery, history of pregnancy termination, height, maternal age at delivery, marital status, and deprivation status. A sensitivity analysis was conducted, additionally adjusting for smoking history and restricting the data to 1992 onwards, as smoking history was not routinely collected before 1992. Multiple imputation by chained equations was used to replace missing values in maternal height, and marital and smoking status.^10^ Smoking status was only imputed from 1992 to 2008. Five imputations were created using a set of appropriate imputation models constructed of all co-variables and outcome variables, stratified by year categories. An additional 35 imputations were created to confirm that the results were robust to the number of imputations used.

The population-attributable fraction (*AF*_p_) attributable to an increase in the number of previous miscarriages for all-cause preterm birth was calculated separately at two time points, 1980 and 2008, using the following equation:


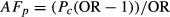
,

where *P*_c_ is the exposure prevalence among cases.

The statistical significance of covariates and interactions in the multiple imputation analyses were assessed using the Wald test. *P* values for all hypothesis tests were two-sided, and statistical significance was set at *P *< 0.05. A Bonferroni correction was used to adjust for multiple interaction tests; *P *< 0.01 was considered indicative of statistical significance. All statistical analyses were performed using stata 12.1 (Stata Corporation, TX, USA).

## Results

A total of 757 351 singleton, live-born first births between 1980 and 2008 were identified, which was reduced to 732 719 after the application of inclusion and exclusion criteria (Figure S1). The maternal characteristics and outcomes were tabulated by number of previous miscarriages (Table1). A greater number of previous miscarriages was associated with older maternal age, shorter gestation, lower deprivation, history of therapeutic pregnancy termination, and current smoking. An increasing number of previous miscarriages was also associated with first births in later years, as well as all preterm birth outcomes, except induced preterm birth at 24–28 weeks of gestation with a diagnosis of pre-eclampsia.

**Table 1:** Descriptive characteristics and outcomes in relation to the number of previous miscarriages within the full cohort, Scotland, 1980–2008

	Number of previous miscarriages										
	All		0		1		2		3+		*P*
*n* (%)	732 719	100.00%	646 382	88.22%	71 660	9.78%	11 308	1.54%	3369	0.46%	–
Height: cm (IQR)**	162	(157–167)	162	(157–167)	162	(157–167)	162	(158–167)	162	(157–167)	<0.001
Age: years (IQR)	25	(21–29)	25	(21–29)	27	(23–31)	28	(24–32)	30	(25–34)	<0.001
Gestation: weeks (IQR)	40	(39–41)	40	(39–41)	40	(39–41)	40	(38–40)	39	(38–40)	<0.001
**Smoking status**: ***n*** %*^,^**											
Non-smoker	234 334	58.03%	203 591	58.08%	25 260	57.67%	4247	58.47%	1236	55.28%	<0.001
Smoker	122 520	30.34%	105 848	30.20%	13 668	31.20%	2221	30.58%	783	35.02%	
Ex-smoker	46 995	11.64%	41 109	11.73%	4874	11.13%	795	10.95%	217	9.70%	
**Socio-economic deprivation status**: ***n*** **%**											
Least deprived, 1	36 552	4.99%	31 831	4.92%	3854	5.38%	668	5.91%	199	5.91%	<0.001
2	98 408	13.43%	86 336	13.36%	9935	13.86%	1661	14.69%	476	14.13%	
3	148 507	20.27%	130 827	20.24%	14 686	20.49%	2303	20.37%	691	20.51%	
4	183 078	24.99%	161 819	25.03%	17 727	24.74%	2731	24.15%	801	23.78%	
5	116 746	15.93%	103 198	15.97%	11 302	15.77%	1733	15.33%	513	15.23%	
6	89 449	12.21%	79 279	12.27%	8516	11.88%	1281	11.33%	373	11.07%	
Most deprived, 7	59 979	8.19%	53 092	8.21%	5640	7.87%	931	8.23%	316	9.38%	
**Marital Status**: ***n*****, %** **											
Married	407 370	61.52%	356 232	60.87%	42 264	65.95%	6850	68.92%	2024	69.58%	<0.001
Other (incl. divorced, cohabiting)	254764	38.48%	228 969	39.13%	21 821	34.05%	3089	31.08%	885	30.42%	
**Therapeutic pregnancy termination**: ***n*** **%**											
None	669 291	91.34%	59 3108	91.76%	63 171	88.15%	9977	88.23%	3035	90.09%	<0.001
History of therapeutic termination	63 428	8.66%	53 274	8.24%	8489	11.85%	1331	11.77%	334	9.91%	
**Year categories**: ***n*** **%**											
1980–1983	101 672	13.88%	92 448	14.30%	7820	10.91%	1094	9.67%	310	9.20%	<0.001
1984–1987	103 693	14.15%	94 093	14.56%	8231	11.49%	1087	9.61%	282	8.37%	
1988–1991	112 981	15.42%	101 420	15.69%	9818	13.70%	1367	12.09%	376	11.16%	
1992–1995	106 212	14.50%	92 968	14.38%	11 060	15.43%	1725	15.25%	459	13.62%	
1996–1999	97 970	13.37%	85 059	13.16%	10 652	14.86%	1691	14.95%	568	16.86%	
2000–2003	88 768	12.11%	76 574	11.85%	9 956	13.89%	1697	15.01%	541	16.06%	
2004–2008	121 423	16.57%	103 820	16.06%	14 123	19.71%	2647	23.41%	833	24.73%	
**Preterm birth outcomes**: ***n*** **%**											
All-cause PTB – all	45 967	6.27%	39 419	6.10%	5084	7.09%	1047	9.26%	417	12.38%	<0.001
24–28 weeks of gestation	2929	0.42%	2397	0.39%	368	0.55%	121	1.17%	43	1.28%	<0.001
29–32 weeks of gestation	7156	1.03%	6074	0.99%	814	1.21%	189	1.81%	79	2.34%	<0.001
33–36 weeks of gestation	35 882	4.97%	30 948	4.85%	3902	5.54%	737	6.70%	295	8.76%	<0.001
Spontaneous PTB – all	30 462	4.25%	26 495	4.18%	3150	4.52%	604	5.56%	213	6.32%	<0.001
24–28 weeks of gestation	1883	0.27%	1552	0.26%	233	0.35%	75	0.73%	23	0.68%	<0.001
29–32 weeks of gestation	3525	0.51%	3042	0.50%	368	0.55%	81	0.78%	34	1.01%	<0.001
33–36 weeks of gestation	25 054	3.52%	21 901	3.48%	2549	3.69%	448	4.18%	156	4.63%	<0.001
Induced PTB with pre-eclampsia – all	4827	0.70%	4158	0.68%	539	0.80%	88	0.85%	42	1.25%	<0.001
24–28 weeks of gestation	392	0.06%	330	0.05%	52	0.08%	9	0.09%	1	0.03%	0.048
29–32 weeks of gestation	1377	0.20%	1193	0.20%	144	0.22%	27	0.26%	13	0.39%	0.008
33–36 weeks of gestation	3058	0.44%	2635	0.43%	343	0.51%	52	0.50%	28	0.83%	<0.001
Induced PTB without pre-eclampsia – all	10 671	1.46%	8761	1.36%	1393	1.94%	355	3.14%	162	4.81%	<0.001
24–28 weeks of gestation	654	0.09%	515	0.08%	83	0.12%	37	0.33%	19	0.56%	<0.001
29–32 weeks of gestation	2250	0.31%	1837	0.28%	300	0.42%	81	0.72%	32	0.95%	<0.001
33–36 weeks of gestation	7767	1.06%	6409	0.99%	1010	1.41%	237	2.10%	111	3.29%	<0.001

IQR, interquartile range; PTB, preterm birth.

* Smoking data was available from 1992 onwards.

** Percentage of data missing: marital status - 70585 (9.6%), height - 106661 (14.6%), smoking history (from 1992 onwards) - 43998 (10.6%).

There were positive associations between previous miscarriage and all preterm birth outcomes in the univariate analysis (Table2). Independent, positive associations were found for all outcomes after adjustment for maternal characteristics. Associations with previous miscarriage, treated as a binary, categorical, and continuous variable, are tabulated along with all adjusted and unadjusted odds ratios. There was a dose–response relationship, as evidenced by stronger associations for women with three or more miscarriages than for women with one or two miscarriages, for each outcome, and by the significant per-unit increase.

**Table 2:** Odds ratios for the association between previous miscarriage and the risk of all-cause preterm birth and preterm birth subtypes

Outcome	Unadjusted odds ratios (95% CI)						Adjusted odds ratios (95% CI)*					
	History of miscarriage	Number of previous miscarriages				*P* ***	History of miscarriage	Number of Previous Miscarriages				*P* ***
		1	2	≥3	Per unit Increase**			1	2	≥3	Per unit Increase**	
Preterm birth (all cause) (*n* = 732 719)	1.26 (1.23–1.30)	1.18 (1.14–1.21)	1.57 (1.47–1.68)	2.18 (1.96–2.41)	1.23 (1.21–1.26)	<0.001	1.26 (1.22–1.29)	1.17 (1.14–1.21)	1.56 (1.46–1.66)	2.14 (1.93–2.38)	1.23 (1.21–1.25)	<0.001
Spontaneous (*n* = 717 214)	1.14 (1.10–1.18)	1.08 (1.04–1.13)	1.35 (1.24–1.47)	1.65 (1.44–1.90)	1.13 (1.11–1.16)	<0.001	1.19 (1.15–1.24)	1.13 (1.09–1.17)	1.45 (1.34–1.58)	1.82 (1.58–2.09)	1.18 (1.15–1.21)	<0.001
Induced (all) (*n* = 702 257)	1.52 (1.46–1.59)	1.36 (1.30–1.43)	2.03 (1.84–2.23)	3.25 (2.81–3.74)	1.42 (1.38–1.46)	<0.001	1.37 (1.31–1.43)	1.25 (1.19–1.31)	1.74 (1.57–1.91)	2.60 (2.25–3.00)	1.31 (1.28–1.35)	<0.001
Induced (pre-eclampsia) (*n* = 691 579)	1.22 (1.13–1.33)	1.18 (1.08–1.29)	1.25 (1.01–1.55)	2.08 (1.53–2.82)	1.19 (1.12–1.26)	<0.001	1.15 (1.06–1.25)	1.12 (1.02–1.23)	1.13 (0.91–1.40)	1.78 (1.31–2.42)	1.13 (1.06–1.20)	<0.001
Induced (no pre-eclampsia) (*n* = 697 423)	1.66 (1.58–1.74)	1.45 (1.37–1.53)	2.40 (2.15–2.67)	3.80 (3.24–4.46)	1.52 (1.47–1.57)	<0.001	1.47 (1.40–1.55)	1.31 (1.24–1.39)	2.00 (1.80–2.23)	2.95 (2.51–3.47)	1.39 (1.34–1.44)	<0.001

* Adjusted for history of pregnancy termination, maternal height, marital status, maternal age at delivery, deprivation status, and year of delivery.

** Number of previous miscarriages was treated as a continuous variable (truncated at three because of the small numbers of women with more than three previous miscarriages).

*** *P* for trend.

**Figure 1:**
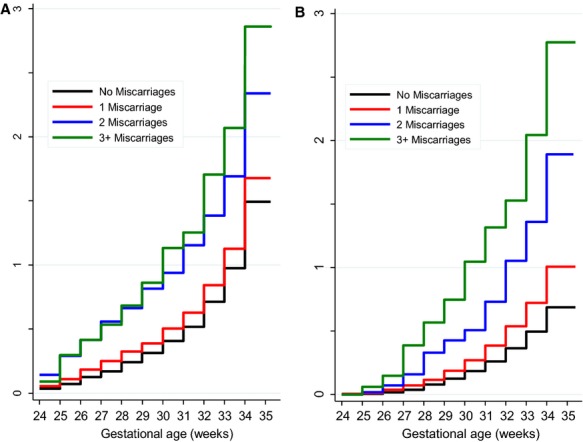
Cumulative incidence of preterm first birth subtypes from 24 weeks of gestation onwards in relation to number of previous miscarriages, Scotland, 1980–2008. Preterm birth subtypes that were not the outcome of interest were not included. The *y*-axis is truncated to 35 weeks of gestation to allow for a better visualization of the differences in incidence of extreme preterm births. A Cumulative incidence of spontaneous preterm first birth in 717 214 nulliparous women. B Cumulative incidence of induced preterm first birth without a diagnosis of pre-eclampsia in 697 423 nulliparous women.

Cox proportional hazards models demonstrated that the relative hazard of preterm birth associated with previous miscarriage differed across the gestational age range of 24–36 weeks of gestation (*P* < 0.001, i.e. the proportional hazards assumption was violated). When analysed by subtype, the risk varied across 24–36 weeks of gestation for spontaneous preterm birth (*P* < 0.001; Figure1A). and induced preterm birth without a diagnosis of pre-eclampsia (*P* = 0.001; Figure1B). In contrast, the association between previous miscarriage and induced preterm birth with a diagnosis of pre-eclampsia did not differ across the range of 24–36 weeks of gestation (*P* = 0.24). Consequently, we stratified the analysis of the relationship between previous miscarriage and the risk of preterm birth by gestational age for the outcomes that violated the proportional hazards assumption (Table3).

**Table 3:** Odds ratios for the association between previous miscarriage and the risk of all-cause preterm birth, spontaneous preterm birth, and induced preterm birth without a diagnosis of pre-eclampsia, stratified by gestational age at delivery

Outcome	Unadjusted odds ratios (95% CI)						Adjusted odds ratios (95% CI)*					
	History of miscarriage	Number of previous miscarriages				*P* ***	History of miscarriage	Number of Previous Miscarriages				*P* ***
		1	2	≥3	Per unit Increase**			1	2	≥3	Per unit Increase**	
**Preterm birth (all-cause)**												
24–28 weeks (*n* = 689 681)	1.69 (1.54–1.86)	1.40 (1.25–1.56)	2.99 (2.49–3.59)	3.69 (2.72–5.00)	1.57 (1.47–1.67)	<0.001	1.73 (1.57–1.90)	1.43 (1.28–1.60)	3.12 (2.59–3.57)	3.87 (2.85–5.26)	1.60 (1.50–1.70)	<0.001
29–32 weeks (*n* = 693 908)	1.36 (1.27–1.45)	1.22 (1.14–1.32)	1.84 (1.59–2.13)	2.67 (2.14–3.35)	1.32 (1.25–1.38)	<0.001	1.36 (1.28–1.46)	1.23 (1.14–1.33)	1.86 (1.60–2.15)	2.68 (2.14–3.36)	1.32 (1.26–1.39)	<0.001
33–36 weeks (*n* = 722 634)	1.20 (1.16–1.24)	1.13 (1.09–1.18)	1.38 (1.28–1.50)	1.87 (1.64–2.13)	1.17 (1.15–1.20)	<0.001	1.18 (1.14–1.22)	1.12 (1.08–1.16)	1.36 (1.25–1.47)	1.81 (1.59–2.06)	1.16 (1.13–1.19)	<0.001
**Preterm birth (spontaneous)**												
24–28 weeks (*n* = 688 635)	1.62 (1.44–1.83)	1.37 (1.19–1.57)	2.86 (2.27–3.61)	3.05 (2.02–4.61)	1.51 (1.40–1.63)	<0.001	1.79 (1.58–2.01)	1.49 (1.30–1.72)	3.33 (2.63–4.21)	3.71 (2.44–5.62)	1.63 (1.50–1.77)	<0.001
29–32 weeks (*n* = 690 277)	1.21 (1.10–1.33)	1.10 (0.99–1.23)	1.58 (1.26–1.97)	2.30 (1.64–3.23)	1.21 (1.13–1.30)	<0.001	1.33 (1.21–1.47)	1.20 (1.08–1.34)	1.83 (1.46–2.28)	2.77 (1.97–3.90)	1.31 (1.22–1.40)	<0.001
33–36 weeks (*n* = 711 806)	1.14 (1.09–1.19)	1.10 (1.05–1.15)	1.29 (1.17–1.43)	1.42 (1.18–1.70)	1.12 (1.09–1.15)	<0.001	1.10 (1.06–1.15)	1.07 (1.02–1.12)	1.24 (1.12–1.37)	1.35 (1.13–1.62)	1.09 (1.06–1.13)	<0.001
**Preterm birth (induced, no pre-eclampsia)**												
24–28 weeks (*n* = 687 406)	2.05 (1.70–2.48)	1.47 (1.17–1.85)	4.25 (3.04–5.94)	7.59 (4.79–12.01)	1.89 (1.69–2.11)	<0.001	1.89 (1.56–2.28)	1.37 (1.09–1.74)	3.79 (2.71–5.32)	6.49 (4.07–10.32)	1.78 (1.59–2.00)	<0.001
29–32 weeks (*n* = 689 002)	1.71 (1.54–1.90)	1.49 (1.32–1.68)	2.61 (2.09–3.26)	3.58 (2.52–5.10)	1.55 (1.44–1.66)	<0.001	1.56 (1.40–1.74)	1.38 (1.22–1.56)	2.27 (1.81–2.84)	2.95 (2.07–4.20)	1.44 (1.34–1.55)	<0.001
33–36 weeks (*n* = 694 519)	1.61 (1.52–1.71)	1.44 (1.34–1.65)	2.19 (1.92–2.49)	3.56 (2.94–4.31)	1.48 (1.42–1.54)	<0.001	1.41 (1.33–1.50)	1.28 (1.20–1.37)	1.79 (1.57–2.05)	2.69 (2.22–3.26)	1.34 (1.28–1.39)	<0.001

* Adjusted for history of pregnancy termination, maternal height, marital status, maternal age at delivery, deprivation status, and year of delivery.

** Number of previous miscarriages was treated as a continuous variable (truncated at three because of the small numbers of women with more than three previous miscarriages).

*** *P* for trend.

A sensitivity analysis restricting the data to 1992 onwards, and additionally adjusting for smoking history, did not materially change the results.

**Figure 2:**
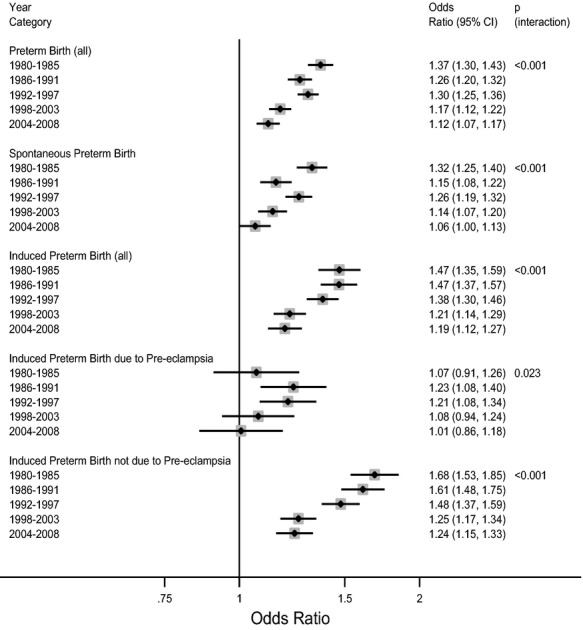
Adjusted odds ratios for preterm first birth subtypes in Scotland from 1980 to 2008, stratified by year categories. Preterm birth subtypes that were not the outcome of interest were not included in the analyses. Odds ratios adjusted for deprivation status, maternal age, marital status, previous and pregnancy termination. The *P* value for interaction is from a Wald test of the null hypothesis that the odds ratios did not significantly differ across the period 1980–2008, where year is treated as a continuous variable. Adjusted odds ratio for a one-unit increase in miscarriage (coded as 0, 1, 2, and 3 or more) in relation to risk of: all-cause preterm first births among 732 719 women; spontaneous preterm first births among 717 214 women; all induced preterm first births among 702 257 women; induced preterm first births with a diagnosis of pre-eclampsia among 691 579 women; and induced preterm first births without a diagnosis of pre-eclampsia among 697 423 women.

The strength of the association between previous miscarriage and all-cause preterm birth progressively weakened over the period 1980–2008 (*P* for interaction < 0.001; Figure2). When analysed by subtype, the trend was observed for all classifications of preterm birth except induced preterm birth with a diagnosis of pre-eclampsia. For this outcome, the *P* value for the interaction was not significant with Bonferroni correction, and there was no clear pattern of change (Figure2).

The population-attributable fraction (*AF*_p_) for all-cause preterm birth attributable to miscarriage was 3.4% in 1980, but decreased to 2.0% in 2008. Concurrently, the proportion of women reporting a history of miscarriage increased from 9.3% in 1980 to 15.8% in 2008.

## Discussion

### Main findings

We found a strong, independent relationship between previous miscarriages and the subsequent risk of preterm birth, and this was evident for all types of preterm birth. The associations were strongest for extremely preterm birth (24–28 weeks of gestation), and this was because of stronger associations with spontaneous preterm birth and preterm births that were induced without a diagnosis of pre-eclampsia. The association between previous miscarriage and preterm birth progressively weakened over the period 1980–2008. This was a result of a weakening in the association between previous miscarriage and both spontaneous preterm birth and preterm births that were induced without a diagnosis of pre-eclampsia. The *AF*_p_ for preterm birth attributable to miscarriage decreased from 3.4 to 2.0% over the study period.

### Strengths and limitations of the study

Strengths of the present study include the large sample size, the prolonged duration of the study, the availability of information to allow some classification of subtypes of preterm birth, and the availability of some covariate data to allow multivariate analysis; however, some important information was missing. First, we lacked information on the management of previous miscarriages (surgical versus non-surgical). Future studies may be able to perform record linkage to assess our interpretation directly, and we hypothesise stronger associations between previous miscarriage and preterm birth where the procedure was managed surgically compared with those managed medically. Furthermore, we lacked information on desirable variables, such as ethnicity and weight.

Second, there is a lack of information on the gestational age at miscarriage in this data set and, to the authors’ knowledge, in any other data set of this magnitude. A large database is necessary for this analysis. Assuming that 12% of the population are exposed, 6% of births are preterm, and an OR of 1.5 in 1980, declining by 5% annually, then a sample size calculation indicates that 80 000 women would be required (for 90% power and *α* = 0.05, two-sided). Larger sample sizes will still be required to study subtypes of preterm birth. Consequently, analyses that include the gestational age at previous miscarriage are unlikely to be possible.

Third, the definition of miscarriage changed during the study period, from the inclusion of losses at <28 weeks of gestation to the inclusion of losses at <24 weeks of gestation after October 1992. In the former period, losses that would subsequently have been classified as preterm births were instead considered miscarriages; however, the majority of miscarriages occur in the first trimester and an estimated 10% occur in the second trimester.^11^ Furthermore, within the current data set from 1993 to 2008 <0.5% of all births occurred during this gestational time frame; therefore, this change in classification is unlikely to impact the results.

Finally, we confined the analysis to nulliparous women to eliminate the complexities of dealing with previous preterm births and miscarriages that occurred following previous births. Future studies may also address the associations in parous women, but highly detailed information would be required for informative analyses.

### Interpretation, in light of other evidence

Previous studies have found associations between miscarriage and a number of obstetric complications, including preterm birth,^11^ as observed in the present study. The association did not appear to be caused by a confounding effect of maternal characteristics, such as advanced maternal age, as it was very similar in univariate and multivariate analyses. Potential explanations for this association include surgical management of miscarriage, which can lead to cervical damage (see below).^4^ The association could, however, also be explained by other factors such as maternal stress,^12^ or by changes in the nature of the previous spontaneous losses.

We found that the association between previous miscarriage and the subsequent risk of preterm birth weakened over the period of study. We have previously described a similar weakening in the association between previous therapeutic pregnancy termination and the subsequent risk of preterm birth in the same population, and over the same period of time.^5^ The weakening in the association with therapeutic pregnancy termination was paralleled by a decline in the use of surgical evacuation without cervical pre-treatment as a method of therapeutic pregnancy termination. We speculated that the decreasing use of methods employing forceful dilation of the cervix led to less cervical damage, and hence to a decline in the subsequent risk of preterm birth.^5^ We speculate that a similar mechanistic link might explain the declining association between previous miscarriage and preterm birth observed in the present study, as there have been comparable changes in the management of incomplete miscarriage. First, prostaglandins are employed to prime the cervix prior to surgical evacuation.^13^ Second, there has been an increasing use of expectant and purely medical management of incomplete miscarriage.^6^

This interpretation is consistent with the observation that the progressive loss of the association was seen for spontaneous preterm birth and induced preterm birth for a reason other than pre-eclampsia. Many of the latter group will have occurred in the context of preterm prelabour rupture of membranes (PPROM). Both spontaneous preterm birth and preterm birth following PPROM are associated with cervical integrity.^14^ In addition, this theory is consistent with the decrease in *AF*_p_ attributable to miscarriage over the study period, in spite of a greater proportion of women reporting a history of miscarriage. This finding is also suggestive of an additional causal factor, such as miscarriage management, decreasing in frequency over time.

## Conclusion

We have shown that the relationship between previous miscarriage and preterm birth declined in Scotland over the period 1980–2008. We previously observed a similar trend for prior history of induced pregnancy termination. Both observations could be explained by the declining use of purely surgical methods for evacuation of the uterus.

### Disclosure of interests

CO, MF, and AW have no financial or non-financial interests that may be relevant to the submitted work. GS reports personal fees outside the submitted work from GlaxoSmithKline: to attend advisory boards; to act as a consultant around novel treatment for preterm birth; and to attend the Scientific Advisory Board on Pre-eclampsia; personal fees from Roche Diagnostics International to attend an Advisory Board meeting in May 2014; and sponsorship from Chiesi Ltd to attend the North Yorkshire Neonatal meeting in February 2014. In addition, GS is a joint holder of a patent (US61/253936: ‘Treatment of preterm neonates’) for a novel treatment for preterm infants currently entering national phase in the USA, Canada, EU, Japan, Australia, and China pending.

### Contribution to authorship

GS formed the hypothesis. MF obtained the data. CO performed the statistical analysis with supervision from GS and AW. CO, AW, and GS drafted the article. All authors critically reviewed the article and approved it for submission. All authors, external and internal, had full access to all of the data (including statistical reports and tables) in the study, and can take responsibility for the integrity of the data and the accuracy of the data analysis.

### Details of ethics approval

Approval for the record linkage was provided by the NHS National Services Scotland Privacy Advisory Committee (PAC).

### Funding

This study was funded by the National Institute for Health Research (NIHR) Cambridge Comprehensive Biomedical Research Centre (Women's Health & Public Health themes) and a Medical Research Council (MRC) PhD fellowship (CO). No funding bodies had any role in the study design, data collection and analysis, decision to publish, or preparation of the article.

## Supporting Information

**Figure S1.** Selection of the study cohort.
