# Supplementary material for: Previous miscarriage and the subsequent risk of preterm birth in Scotland, 1980–2008: a historical cohort study
Source: BJOG. 2015 Jan 28;122(11):1525–34. doi: 10.1111/1471-0528.13276 (PMC4611958; doi:10.1111/1471-0528.13276)
Supplement: Supplementary file 1 — Figure S1. Selection of the study cohort. [file bjo0122-1525-sd1.pdf]

Figure S1: Selection of the study cohort

757,351 singleton live-born first deliveries (1980-2008)

Exclusions: n = 2611

Neonatal deaths due to congenital malformation or Rhesus disease (n=1,651, 0.22%)
Gestational age: <24 & >43 weeks (n=467, 0.06%)
Birth-weight: <500g (n=269, 0. 04%)
Maternal age: less than 13 or greater than 45 (n=119, 0.02%)
Height: less than 100cm or greater than 250cm (n=105, 0.01%)

Missing Data: n = 22021

Gestational age (n= 2,471, 0.33%)
Birth-weight (n=494, 0.06%)
Sex (n= 33, 0.004%)
Miscarriage (n=1, 0.0001%)
Spontaneous delivery (n= 309, 0.04%)
Deprivation Status (n= 18,713, 2.46 %)

732,719 singleton live-birth first deliveries (1980-2008) included in analysis
